# Primary Sjogren’s Disease Coexisting With Myasthenia Gravis: Two Distinct Autoimmune Diseases Successfully Treated With a Rituximab-Based Induction Regimen

**DOI:** 10.7759/cureus.78018

**Published:** 2025-01-26

**Authors:** Abraham Mohan, Lijo James, Aiswarya Mohan, Thomas Mathew, Sojan K Scaria

**Affiliations:** 1 Rheumatology, Caritas Hospital and Institute of Health Sciences, Kottayam, IND; 2 Neurology, Caritas Hospital and Institute of Health Sciences, Kottayam, IND; 3 Research and Development, Caritas Hospital and Institute of Health Sciences, Kottayam, IND; 4 Ophthalmology, Caritas Hospital and Institute of Health Sciences, Kottayam, IND; 5 Internal Medicine, Caritas Hospital and Institute of Health Sciences, Kottayam, IND

**Keywords:** autoimmune diseases, central pontine myelinolysis, myasthenia gravis, primary sjogren's syndrome, rituximab

## Abstract

Primary Sjögren’s syndrome (pSS) and myasthenia gravis (MG) are autoimmune diseases and are rarely reported in coexistence. MG is a chronic autoimmune neuromuscular disease where antibodies bind to acetylcholine receptors or to functionally related molecules in the postsynaptic membrane, weakening the skeletal muscles and causing diplopia, ptosis, and difficulty in breathing and swallowing. In this report, we discuss the case of a 43-year-old female patient who presented with dry eyes, weight loss, fatigue, ptosis, dysarthria, and quadriparesis, which ultimately led to the diagnosis of pSS and MG. Severe dry eyes, myasthenic symptoms, hypokalemic paralysis, and pancytopenia were among the disease and treatment-related consequences that had to be managed during the course of treatment. The symptoms of MG and pSS were initially managed with high-dose corticosteroids, azathioprine, and pyridostigmine/neostigmine. Escalation to an induction regimen based on rituximab was required due to persistent disease activity of both MG and pSS. Significant hematological and clinical improvements were noted after treatment, highlighting rituximab's effectiveness in inducing remission in these overlapping autoimmune diseases. To the best of our knowledge, this is the first case in the literature where these coexisting diseases were simultaneously, successfully treated with a rituximab-based induction regimen.

## Introduction

Autoimmune diseases are conditions in which your immune system mistakenly damages healthy cells in your body. Sjögren's syndrome (SS) is an autoimmune disease that mostly affects the exocrine and salivary glands and has a 10-60% chance of brain involvement that is typically disregarded [1]. It may occur in two forms, primary and secondary, which is associated with another autoimmune disease, most commonly rheumatoid arthritis. As SS progresses through its pathological course, clinical symptoms appear gradually. Lacrimal hypofunction (xerophthalmia) and dry mouth (xerostomia) due to hyposalivation, which are caused by self-perpetuating immune-mediated loss of acinar and ductal cells of the lacrimal and salivary glands, are frequently the initial signs of primary SS (pSS) [2]. There are a number of extra-glandular manifestations in SS including neurological, renal, rheumatological, vascular, gastric, and pulmonary symptoms [3]. As, currently, there is no single diagnostic tool to confirm the disease, it is challenging to diagnose SS early and accurately. Early diagnosis and timely treatment depend on the appropriate use of trustworthy diagnostic systems.

Myasthenia gravis (MG) is a chronic autoimmune neuromuscular disease. This disease is mediated by a type-II antibody reaction in which antibodies attach to acetylcholine receptors or to functionally related molecules in the postsynaptic membrane, weakening the skeletal muscles and causing diplopia, ptosis, and difficulty breathing and swallowing [4]. The mainstay of treatment in MG involves cholinesterase enzyme inhibitors and immunosuppressive agents [5,6]. New medications for MG include compounds that target plasmablasts, B cells, complement inhibitors, and antagonists of the neonatal fragment crystallizable receptor (FcRn) [7].

The coexistence of pSS and MG in a single patient is exceptionally rare. The coexistence of these two conditions gives rise to unique challenges, as both diseases may present with overlapping and compounding symptoms. Here, we highlight the case of a patient diagnosed with pSS and MG, who achieved significant clinical improvement with a rituximab-based induction regimen.

## Case presentation

A 43-year-old woman presented to the emergency department on October 3, 2022, with multiple episodes of vomiting for two days, weakness in all four limbs with slurring of speech for one day, and decreased responsiveness. She had a history of dry eyes for eight years, generalized weakness, and weight loss for a few months. The patient was initially evaluated at an outside hospital, where she was detected to have severe hypokalemia. An antinuclear antibody (ANA) blot was sent, which was strongly positive for SSA-Ro 52 and SSA-Ro 60 antibodies. She was suspected to have a connective tissue disease and was referred to our tertiary care center.

**Figure 1:**
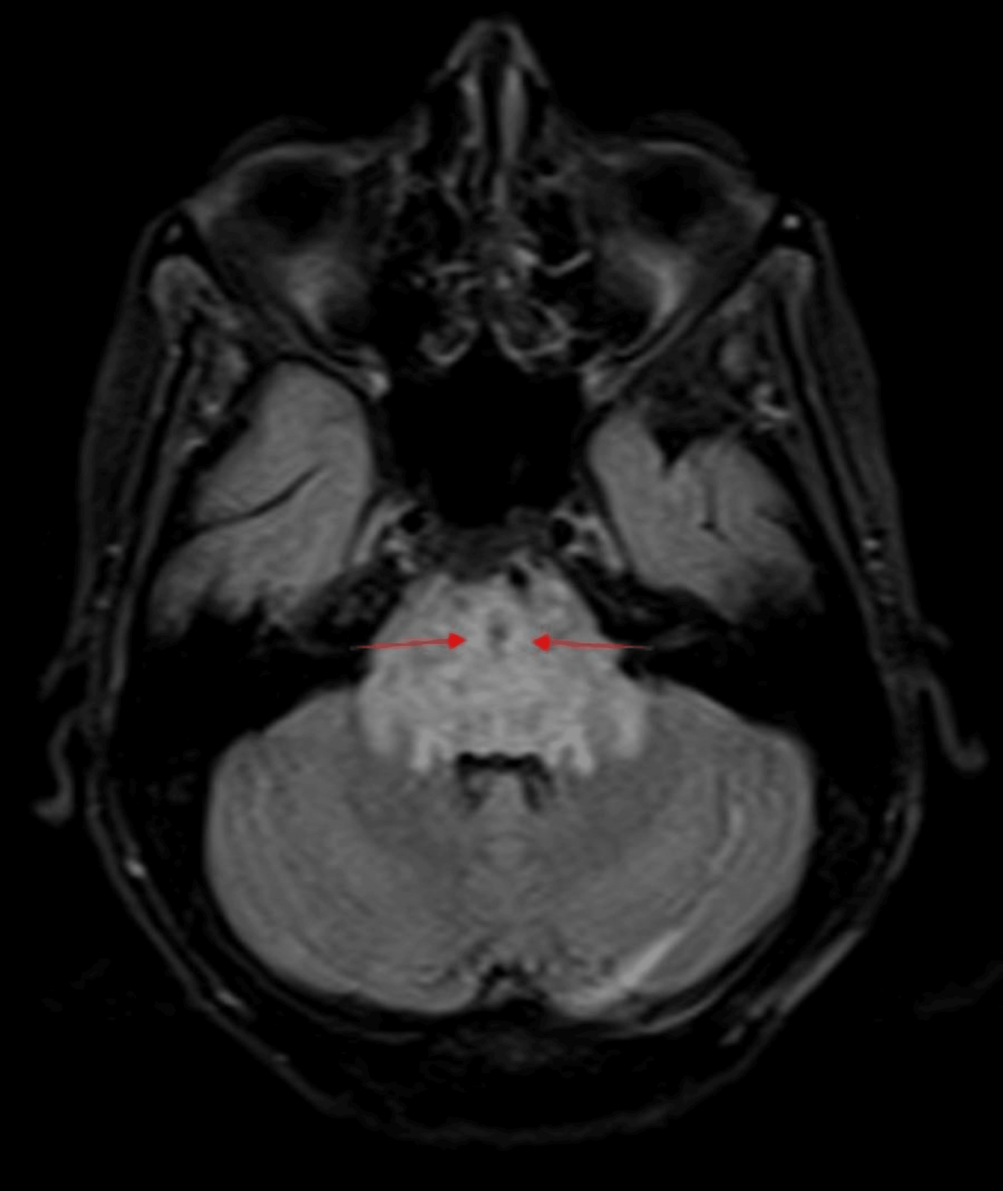
T2/FLAIR image showing swollen pons with hyperintense signals in central pons. There is perifocal edema involving the midbrain, posterior limb of the internal capsule superiorly, medulla inferiorly, and middle cerebellar peduncles posterolaterally. FLAIR: fluid-attenuated inversion recovery

On clinical evaluation here in the emergency room, the patient was drowsy and her serum potassium was 1.4 meq/L. She was admitted to the intensive care unit and started on intravenous potassium chloride correction for severe hypokalemia. The patient became alert in a few hours. On physical examination, the patient had bilateral ptosis with grade 4 muscle power in all four limbs. There was hyporeflexia in the upper limbs. Dysarthria was also present. Her rheumatoid factor was elevated at 69.1 IU/mL. She was suspected to have pSS with hypokalemic paresis. However, bilateral ptosis and dysarthria are not features of hypokalemic paresis. Hence, an MRI brain was done to rule out brain stem involvement, which showed swollen pons with demyelination and patchy hemorrhagic areas with perifocal edema (Figure 1).

The differential diagnoses thought at this point were central pontine myelinolysis and pontine glioma. Common causes of central pontine myelinolysis such as hyponatremia, alcoholism, malnutrition, and inappropriate sodium correction were ruled out. Central pontine myelinolysis can be a rare manifestation of SS; hence, it was attributed to it. In subsequent days, the patient’s ptosis showed fluctuation, and she developed asymmetric ptosis. The patient's acetylcholine receptor antibodies were positive, 2.28 nmol/l (positive > 0.5). With these reports, ptosis and dysarthria were attributed to myasthenia, and the patient was diagnosed with MG. Her MG Activities of Daily Living (MG-ADL) score was 11 at this point and was started on intravenous dexamethasone.

Arterial blood gas analysis showed a low blood pH, 7.218, suggestive of systemic acidosis with low pCO2, 24.5 mmHg. Her serum bicarbonate levels were low at 9.6 mmol/L, suggestive of metabolic acidosis. The patient had a normal anion gap (10.8 mmol/L). She was diagnosed to have normal anion gap metabolic acidosis. Her urine pH was 6.0. Due to the presence of a urine pH>5.5, normal anion gap metabolic acidosis, and hypokalemia, she was diagnosed to have type 1 (distal) renal tubular acidosis. An ophthalmology evaluation was done, including Schirmer's test, which was suggestive of severe dry eyes. The patient had persistent leukocytosis. A peripheral smear was done, which showed neutrophilic leukocytosis with toxic granules. The patient was suspected to have an infection. Blood cultures and urine cultures were sent, but reports were negative. The patient’s hemoglobin levels and platelet counts were normal at this juncture. Her serum creatinine was mildly elevated on admission, 1.3 mg/dl, which became normal in a few days. Ultrasonography of salivary glands showed bilateral swollen parotid and submandibular glands, which was thought to be due to SS. Two-dimensional echocardiography was done, which was normal. CT thorax was done to rule out thymoma, and there was no evidence of any mediastinal mass or any other significant findings.

With the presence of swollen salivary glands, severe dry eyes, type 1 renal tubular acidosis, central pontine myelinolysis, and positive Ro-SSA antibodies, a diagnosis of pSS coexisting with MG was made. The patient satisfied the American College of Rheumatology/European League Against Rheumatism (ACR/EULAR) classification criteria for SS. She was converted to oral potassium supplements once her serum potassium was stable. The patient was started on hydroxypropyl methylcellulose eye drops and gel for eye lubrication and was discharged on October 18, 2022. She was continued on tablet prednisolone 20 mg once daily and pyridostigmine 60 mg 1-1-1 on discharge, with potassium supplementation. Hydroxychloroquine was not started for SS in view of possible drug-induced worsening of MG.

In her three-month follow-up in January 2023, azathioprine tablets were added to oral corticosteroids as the patient had persistent muscle weakness (Grade 4 muscle power in proximal muscles in all four limbs), fatigue, and new-onset diplopia. She was detected to have right lateral rectus palsy. The prednisolone tablet dose was also increased to 40 mg once daily and neostigmine 15 mg thrice daily was added; pyridostigmine was continued at the same dose. Central pontine myelinolysis showed near complete resolution on the follow-up MRI Brain. She was continued on azathioprine plus steroids on the subsequent follow-ups.

On June 26, 2023, the patient presented to the emergency room with complaints of myalgias and fatigue for four days and loose stools for two days. On evaluation, she was detected to have pancytopenia. Hemoglobin (Hb) was 8.6 g/dl, total count (TC) 3450/cu.mm, platelet count 65,000/cu.mm. Azathioprine was stopped because of possible drug-induced pancytopenia. Serum lactate dehydrogenase (LDH) was elevated at 830 U/l. Serum creatine phosphokinase (CPK) was normal at 23 U/l. The patient was suspected to have a secondary infection. Blood culture, Leptospira IgM, and Dengue IgM were negative, and other common infections were ruled out. The patient was, however, given a course of antibiotics. By July 5, 2023, Hb and TC gradually improved to 10.2 g/dl and 8900/cu.mm, respectively, along with other symptoms, but the patient developed severe thrombocytopenia with a platelet count of 31,000/cu.mm, which persisted for the next two weeks. The patient's urine analysis done on July 5, 2023, was normal with no urine albumin, but the urine protein creatinine ratio was raised to 2.4. Her 24-hour urine protein was 598 mg/24 hours. As urine albumin was negative, it was thought to be non-albuminuric proteinuria, which was a result of active SS. EULAR Sjögren's syndrome disease activity index (ESSDAI) at this point was 14.

Table 1 shows the summary of the investigations done over time.

**Table 1:** Summary of laboratory investigations done over time

Investigation	Date	Reference range	Patient value
Serum sodium	October 3, 2022	136-145 meq/L	138 meq/L
Serum potassium	October 3, 2022	3.5 -5.1 meq/L	1.4 meq/L
Rheumatoid factor	October 3, 2022	0-18 IU/ ml	69.1 IU/ ml
ANA blot	October 3, 2022		SSA-Ro 52- 3 + SSA-Ro 60- 3+
Arterial Blood Gas Analysis	October 4, 2022		
pH		7.35-7.45	7.218
pCO2		35-45 mm Hg	24.5 mm Hg
Serum bicarbonate	October 4, 2022	20-30 mmol/L	9.6 mmol/L
Urine pH	October 4, 2022	5-8	6.0
Serum creatinine	October 4, 2022	0.52-1.04 mg/dl	1.3 mg/dl
Acetylcholine receptor antibodies (AChR)	October 10, 2022	Positive > 0.5; Borderline: 0.4-0.5; Negative < 0.4	2.28 nmol/L
Hemoglobin	June 26, 2023	12-15 g/dl	8.6 g/dl
	July 5, 2023		10.2 g/dl
Total count	June 26, 2023	4,000-11,000/cubic mm	3450/cubic mm
	July 5, 2023		8900/cubic mm
Platelet count	June 26, 2023	1.5-4 lakhs/cubic mm	65,000/cubic mm
	July 5, 2023		31,000/cubic mm
Urine protein creatinine ratio	July 5, 2023	0-0.3	2.4
24-hour urine protein	July 6, 2023	30-140 mg/24 hours	598 mg/24 hours

In view of active SS with severe dry eye, severe thrombocytopenia, non-albuminuric proteinuria, and active MG, the patient was planned to give rituximab infusions for induction of disease remission of both diseases. She was given the first dose of rituximab, a 1-gram infusion, on July 11, 2023. Her immunosuppressant was changed from azathioprine to mycophenolate mofetil, starting at 500 mg twice daily on July 12, 2023. The patient had persistent severe dry eye; hence, cyclosporin eye drops were added to hydroxypropyl methylcellulose eye drops and gel. She was discharged on July 13, 2023.

Her second dose of rituximab, 1 gram, was given two weeks later. All her symptoms gradually improved. Severe dry eye and fatigue reduced. Hb and platelet count became normal in two months. The patient’s upper and lower limb proximal muscle weakness due to myasthenia improved. Neostigmine tablets were stopped, and pyridostigmine tablets were tapered to 60 mg, half a tablet twice daily. Oral prednisolone was gradually tapered and stopped by April 2024. She was continued on potassium citrate tablets, 10 mg, twice daily for hypokalemia due to distal renal tubular acidosis. In June 2024, almost one year after the second presentation, the patient had worsening proximal muscle weakness of the lower limbs. Mycophenolate mofetil dose was escalated to 1 gram twice daily, and the pyridostigmine tablet dose was escalated to 60 mg thrice daily. The patient is currently better. Her latest ESSDAI was 0 and MG-ADL was 1.

## Discussion

The co-occurrence of MG and pSS is uncommon. In the current case, the patient presented with clinical features indicative of both pSS and MG, including severe dry eyes, fatigue, quadriparesis, central pontine myelinolysis, thrombocytopenia, dysarthria, bilateral ptosis, and right lateral rectus palsy [4,8]. The clinical features alongside serological evidence of anti-Ro/SSA antibodies and imaging findings of swollen salivary glands confirmed the diagnosis of pSS [9-11]. This patient also had central pontine myelinolysis, which can be a rare manifestation of SS [12-14]. This case highlights that clinicians need to be aware of this fact.

The diagnosis of pSS in our patient was made as per the 2016 ACR/EULAR classification criteria for SS [15]. Labial salivary gland biopsy was not done for this patient, as she satisfied the ACR/ EULAR criteria without biopsy and had numerous extra-glandular manifestations of SS. The pSS is a B-cell-mediated systemic autoimmune illness, characterized by a number of antibodies, such as ANA, anti-SSA (Ro), and anti-SSB (La). There may be a comparable immunologic process between these two diseases that involves distinct targets. The patient was first treated with azathioprine and corticosteroids, which produced some alleviation but also had side effects, such as pancytopenia.

Later, the patient responded well to rituximab. Rituximab is a chimeric monoclonal antibody targeting CD20-positive B cells and has emerged as a promising therapeutic option in this context [16]. By depleting circulating B cells, rituximab reduces the production of autoantibodies, addressing the underlying pathology in both pSS and MG, which was similar to other studies [16-18]. Rituximab proved to be effective in a systematic review of 169 MG patients [18]. The Modified Myasthenia Gravis Foundation of America postintervention scale of minimal manifestations (MM) or better occurred in 44%. MM or better was achieved in 30% of anti-AChR-positive MG and in 72 % of anti-muscle specific kinase antibody-positive MG. Rituximab dramatically reduced systemic disease activity in pSS patients with high ESSDAI scores [19, 20]. This is consistent with the remission our patient experienced.

Mycophenolate mofetil was also given to this patient as a maintenance therapy at a dosage of 500 mg twice daily along with the rituximab induction regimen. Almost a year after starting rituximab, our patient's MG symptoms returned, requiring an increase in mycophenolate mofetil and pyridostigmine dosages. A maintenance dose of rituximab was not given, because the patient was already receiving a maintenance dosage of mycophenolate mofetil and was doing well on it. On relapse of symptoms, mycophenolate mofetil was escalated to an induction dosage of 1 gram twice daily and the patient responded well to this. This emphasizes the necessity of ongoing observation and customized immunosuppressive treatments.

In the current case, the patient showed remarkable clinical improvement following a rituximab-based induction regimen, suggesting its potential as a dual-purpose therapeutic agent for coexisting MG and pSS.

## Conclusions

This case underscores the complexity of managing coexisting distinct autoimmune diseases. Rituximab proved to be a very successful treatment, allowing notable clinical improvement and improving the quality of life of the patient. Further research is required to validate rituximab's dual effectiveness in treating overlapping autoimmune disorders like pSS and MG. Larger randomized, placebo-controlled clinical trials are also needed to further evaluate the clinical efficacy and safety of rituximab therapy for pSS.
